# Hepatitis B Vaccination Induced TNF-*α*- and IL-2-Producing T Cell Responses in HIV− Healthy Individuals Higher than in HIV+ Individuals Who Received the Same Vaccination Regimen

**DOI:** 10.1155/2018/8350862

**Published:** 2018-02-27

**Authors:** Kriangkrai Chawansuntati, Kanokporn Chaiklang, Romanee Chaiwarith, Jutarat Praparattanapan, Khuanchai Supparatpinyo, Jiraprapa Wipasa

**Affiliations:** ^1^Research Institutes for Health Sciences, Chiang Mai University, Chiang Mai 50202, Thailand; ^2^Department of Medicine, Faculty of Medicine, Chiang Mai University, Chiang Mai 50202, Thailand

## Abstract

We investigated cytokine production and expression of degranulation marker CD107a after different strategies of hepatitis B virus (HBV) vaccination in human immunodeficiency virus-infected individuals, which were three doses of 20 *μ*g (standard dose group), four doses of 20 *μ*g (four doses group), or four doses of 40 *μ*g (four double doses group), compared to standard dose vaccination in healthy controls. PBMCs collected at different time points were stimulated *in vitro* with recombinant hepatitis B surface antigen and analyzed by flow cytometry. There was an increase in TNF-*α* production of total and memory CD4+ T cells at 7 months after vaccination in healthy controls compared to the HIV+ group, which received the same standard vaccination regimen. An increase in the IL-2-producing memory CD4+ T cells in the healthy control group was also observed at 7 months after vaccination. No differences were observed between the healthy controls and both groups of four doses at any time point of study. These results suggest that the standard HBV vaccination schedule might induce better production of TNF-*α* and IL-2 from CD4+ T cells in healthy individuals. Modification of HBV vaccination schedule by increasing the frequency and/or dosage may improve the CMI response in HIV-infected individuals. This trial is registered with NCT1289106.

## 1. Introduction

Hepatitis B virus (HBV), which causes hepatitis B liver diseases, is one of the most serious infectious viruses. As HBV and human immunodeficiency virus (HIV) share similar routes of transmission, coinfection with HBV is more common in HIV-infected (HIV+) individuals than in the general population [1–3]. The reported prevalence of HBV/HIV coinfection ranges between 8.7% and 10.4% in Asia, including Thailand [4, 5]. Progression of HBV-related liver diseases in HIV+ individuals is more accelerated than in patients with chronic HBV infection alone [6, 7]. Therefore, prevention of HBV infection by vaccination in HIV+ individuals is strongly recommended [8–10]. However, the efficacy of HBV vaccine to induce protective antibody levels in HIV+ individuals is low compared to that in the HIV− general population [11–16]. Several studies have reported improvement in the response to HBV vaccine by using higher doses [17, 18], increasing the frequencies [18, 19], and increasing both the doses and the frequencies [11, 18, 20].

Besides antibodies that play a crucial role in protection against HBV infection, T cell immune response is considered necessary for sufficient control and clearance of HBV [21]. CD4+ T cell response in the acute phase of self-limiting infection patients is significantly more frequent, strong, and multispecific than that observed in chronic patients [22–24]. These CD4+ T cells have a preferential Th-1 profile, which is specific to multiple epitopes of HBV antigens [24–27]. CD8+ T cells serve a dual function in HBV control, which is the elimination of HBV-infected cells via their cytotoxicity and antiviral activity by secreting antiviral cytokines such as interferons (IFNs) and tumor necrosis factor- (TNF-) *α* without cytolytic activity [28–30].

In contrast to widely studied serological response, CMI response to HBV vaccination is less well understood, especially in HIV+ populations. The precise role of CMI responses in protection against the HBV infection is not yet known. Nevertheless, vaccination with HBV vaccine induced long-lived CMI, which can be boosted by revaccination despite the absence of protective levels of antibodies [31, 32]. This study aimed to investigate the cytokine production and expression of the degranulation marker (CD107a) of T cells in response to *in vitro* recombinant hepatitis B surface antigen (HBsAg) stimulation in HIV+ individuals who received different strategies of HBV vaccination compared to standard vaccination in healthy controls. The results from this study may provide elementary knowledge for addressing important issues for development of an HBV vaccine strategy, especially for HIV+ populations.

## 2. Materials and Methods

### 2.1. Study Population

Participants in this study were the same individuals as those in the study on immunogenicity and safety of different regimens of hepatitis B vaccination in HIV-infected adults reported previously [18]. The individuals consisted of 132 HIV+ and 40 HIV− healthy individuals over eighteen years old, who were seronegative for HBsAg, antibody to hepatitis B surface antigen (anti-HBs), antibody to hepatitis B core antigen (anti-HBc), and antibody to hepatitis C virus (anti-HCV), and without a history of previous HBV vaccination; they were enrolled at Maharaj Nakorn Chiang Mai Hospital, Chiang Mai, Thailand, between February 2011 and May 2012. HIV+ individuals were eligible to participate if they had absolute number of CD4+ T cells of more than 200 cells/mm^3^, had HIV viral load of less than 50 copies/mL, and received antiretroviral therapy (ART). Exclusion criteria included being pregnant or breastfeeding; having a history of hypersensitivity to any component of the vaccine or other immunocompromised conditions besides HIV, renal insufficiency, or decompensated cirrhosis; and receiving chemotherapy or radiation for active malignancy treatment or immunosuppressiveness, or immunomodulating treatment in the last six months before the screening visit. Healthy controls were confirmed HIV negative by using a rapid immunochromatographic screening assay (Pacific Biotech, Thailand).

This study was approved by the ethics committees of the Research Institute for Health Sciences and the Faculty of Medicine, Chiang Mai University, Thailand. All participants were informed about the details of the study and given time to put across any enquiries they had before enrolling in the study consentingly. The authors confirm that all related trials for this intervention were registered to the ClinicalTrials.gov (NCT1289106) on February 1, 2011.

### 2.2. Study Procedure

**Figure 1:**
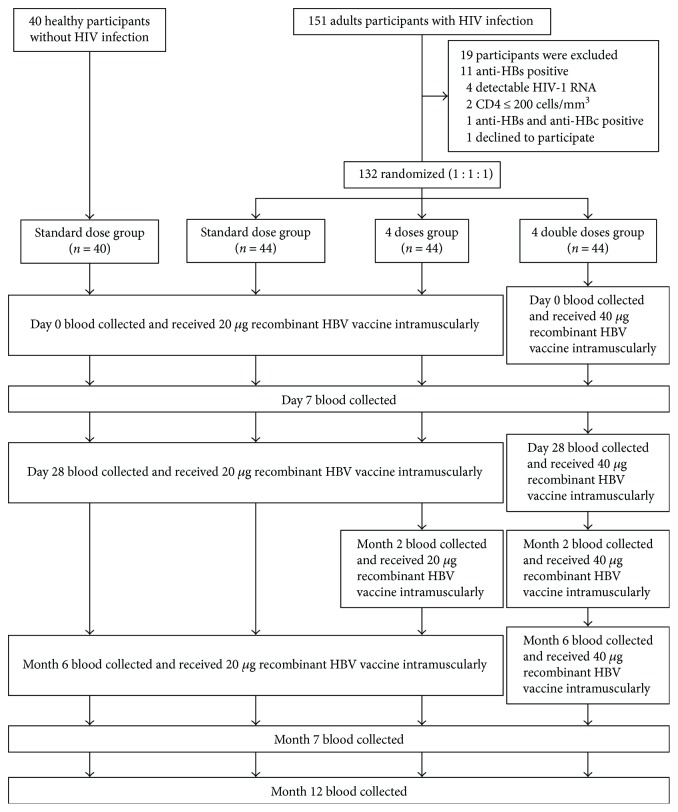
CONSORT diagram of participants in this study.

HIV+ individuals were randomized (1 : 1 : 1) by blocks of six into three groups (44 subjects per group). They were given different doses and frequencies of Hepavax-Gene® vaccine (Berna Biotech Korea Corp, South Korea) containing noninfectious inactivated recombinant HBsAg. Group 1 was defined as the “standard dose group”: the subjects were vaccinated with 20 *μ*g of HBV vaccine at days 0 and 28 and month 6. Group 2 was the “four doses group”: the subjects were vaccinated with 20 *μ*g of HBV vaccine at days 0 and 28 and months 2 and 6. Group 3 was the “four double doses group”: the subjects were vaccinated with 40 *μ*g of HBV vaccine at days 0 and 28 and months 2 and 6. The healthy individuals were vaccinated with the standard regimen similar to that of the HIV+ participants in group 1. Venous blood was collected from all the participants at day 0 (D0) (as baseline), day 7 (D7), day 28 (D28), month 2 (2m) (only for the four doses and four double doses groups), month 6 (6m), month 7 (7m), and month 12 (12m). The CONSORT diagram of participants is shown in Figure 1.

Baseline demographic and clinical characteristics of HIV+ participants have been reported previously [18]. Briefly, the mean age of the HIV+ standard dose group, the HIV+ four doses group, the HIV+ four double doses group, and the HIV− healthy control group was 41.0 ± 6.3, 42.2 ± 7.6, 41.0 ± 6.2, and 33.2 ± 9.5, respectively. The medians of the absolute CD4+ T cell counts of the HIV+ standard dose group, the HIV+ four doses group, and the HIV+ four double doses group were 400 (interquartile range (IQR) 314–558) cells/mL, 544 (IQR 416–731) cells/mL, and 544 (IQR 410–642) cells/mL, respectively. At month 7 after vaccination, the percentages of seroresponders (anti-HBs ≥ 10 mIU/mL) were 88.6% in the HIV+ standard dose group, 93.2% in the HIV+ four doses group, 95.4% in the HIV+ four double doses group, and 94.7% in the HIV− healthy control group.

### 2.3. Isolation of Peripheral Blood Mononuclear Cells

PBMCs were isolated by gradient centrifugation, as previously described [33]. Participants' whole blood was diluted with plain RPMI 1640 medium (Life Technologies, USA), and Ficoll-Hypaque solution (Biochrom, Germany) was layered under the diluted blood. After centrifugation, the PBMC enriched interface was collected, and the residual red blood cells were lysed using ammonium-chloride-potassium lysis buffer. The PBMCs were resuspended in fetal bovine serum (FBS; Biochrom, Germany) containing 10% dimethyl sulfoxide (DMSO; Sigma-Aldrich, USA) and stored in liquid nitrogen until use.

### 2.4. Cell Stimulation and Determination of Cytokine-Producing T Cells

Cryopreserved PBMCs from each participant at all time points were investigated simultaneously. After overnight rest of thawed PBMC, the cells were cultured in the presence of anti-CD107a-PE in complete RPMI containing 2 *μ*g/mL of recombinant HBsAg (MyBioSource, USA) and 5 *μ*g/mL of PHA (Sigma-Aldrich, USA) as the positive stimulation control or complete media alone as the unstimulated control at 37°C in a 5% CO_2_ incubator for 16–18 hr.

Cytokine-producing T cells analysis was performed as described previously [34]. Briefly, the overnight culture was further incubated with 5 *μ*g/mL of brefeldin A and 1 *μ*M of monensin (Sigma-Aldrich, USA) for 4 hr. Then, the cells were stained with anti-CD8-PE Alexa Fluor 610 (Life Technologies, USA), and anti-CD4-APC/Cy7 and anti-CD45RO-Pacific Blue (BioLegend, USA). After fixation and permeabilization, intracellular staining was performed by incubating the cells with anti-CD3-Krome Orange (Beckman Coulter, USA), and with anti-TNF-*α*-FITC, anti-IFN-*γ*-PerCP/Cy5.5, anti-IL-2-PE/Cy7, and anti-IL-10-APC (BioLegend, USA). At least 100,000 lymphocytes were collected for each sample by using Cyan ADP 9-color flow cytometer (Beckman Coulter, USA). Flow cytometric analysis of cytokine-producing or degranulation maker CD107a-expressing T cells was performed by using Kaluza software (Beckman Coulter, USA).

### 2.5. Flow Cytometric Analysis

To assess cytokine production and CD107a expression by CD4+ and CD8+ T cells, T cells were identified from the CD3+ population by Boolean combination gating of forward scatter (FSC) versus side scatter (SSC) plot and CD3 versus SSC plot. Single-positive CD4+ and CD8+ populations were then identified within the CD3+ gate. To evaluate cytokine production and degranulation marker of CD4+ or CD8+ populations, IFN-*γ*, IL-2, IL-10, and CD107a were plotted against TNF-*α*.

**Figure 2:**
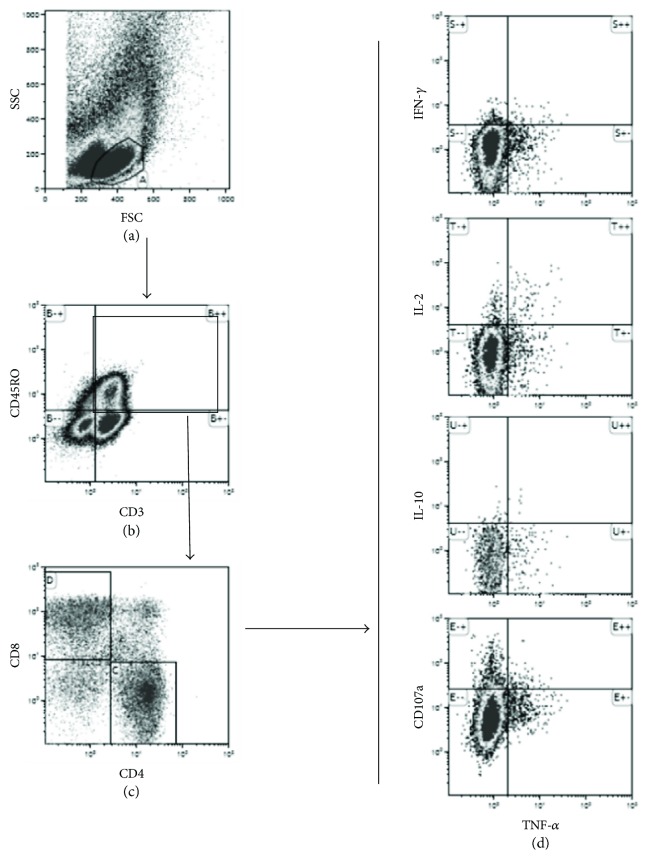
Gating strategies for flow cytometric analysis. The gating strategy used to determine cytokine-producing and CD107a-expressing memory T cells (a–c). Memory T cells were identified from lymphocyte population (a) and double positive population of CD3 versus CD45RO plot (b). Memory CD4+ or CD8+ T cells were then identified from CD4 versus CD8 plot (c). Cytokine production and expression of surface CD107a of CD45RO+CD4+ or CD8+ memory T cell subpopulation were then evaluated (d).

To assess cytokine production and CD107a expression by memory T cells, CD45RO was used as memory marker of T cells (Figures 2(a)–2(d)). Lymphocyte population was initially determined from plot of FSC versus SSC (Figure 2(a)). Memory T cells (CD3+CD45RO+) were then gated from CD3 versus CD45RO plot (Figure 2(b)). Single-positive CD4+ or CD8+ populations were then identified from CD4 versus CD8 plot (Figure 2(c)). Expression of intracellular cytokines or surface CD107a of memory T cells were identified from plots of IFN-*γ*, IL-2, IL-10, and CD107a against TNF-*α* (Figure 2(d)).

### 2.6. Statistical Analysis

All data sets were assessed for normal distribution by using D'Agostino and Pearson omnibus normality test. Paired *t*-test or Wilcoxon signed-rank test and independent *t*-test or Mann–Whitney *U* test were then used depending on data distribution of matched or independent data, respectively. A comparison between study groups was assessed by using Kruskal-Wallis test. Statistical analysis was done by using GraphPad Prism Version 5 software (GraphPad Software, USA). A *p* value of less than 0.05 was considered statistically significant.

## 3. Results

### 3.1. Cytokine Production and CD107a Expression of CD4+ and CD8+ T Cells

Vaccination and blood collection schedules carried out in this study are presented as a CONSORT diagram in Figure 1. Peripheral blood mononuclear cells (PBMCs) were isolated for determination of immunological responses. Cytokine-producing and CD107a-expressing CD4+ or CD8+ T cells were identified, as described in Materials and Methods. The results are presented as fold increases of the percentages of CD4+ or CD8+ T cells that produced TNF-*α*, IFN-*γ*, IL-2, and IL-10 or expressed CD107a in response to *in vitro* recombinant HBsAg stimulation over the medium alone. The fold increases of the percentages of cytokine-producing and CD107a-expressing CD4+ and CD8+ T cells in each of the study groups were compared between D0 as the baseline, D7, 1m, 2m (only for the four doses and four double doses groups), 6m, 7m, and 12m after vaccination. In the healthy control group, only the median fold increase of TNF-*α*-producing CD4+ T cells in response to recombinant HBsAg stimulation *in vitro* at 7m was statistically significantly higher than that at D0 (*p* = 0.02), D7 (*p* = 0.005), 6m (*p* = 0.033), and 12m (*p* = 0.019) after vaccination (data not shown). No differences in the median fold increase of the TNF-*α*-producing CD8+ T cells and the IFN-*γ*-, IL-2-, and IL-10-producing and CD107a-expressing CD4+ and CD8+ T cells between all the time points of the study were observed in this study group. There were no statistical differences between the median fold increases of the cytokine-producing and CD107a-expressing CD4+ and CD8+ T cells between the time points in all the three HIV+ study groups. In response to positive control phytohaemagglutinin (PHA) stimulation, strong cytokine production and CD107a expression were observed in all the participants regardless of their HIV status. There were no differences in these responses between the groups at all the time points of the study (data not shown).

### 3.2. Cytokine Production and CD107a Expression of CD4 and CD8 Memory T Cells

**Figure 3:**
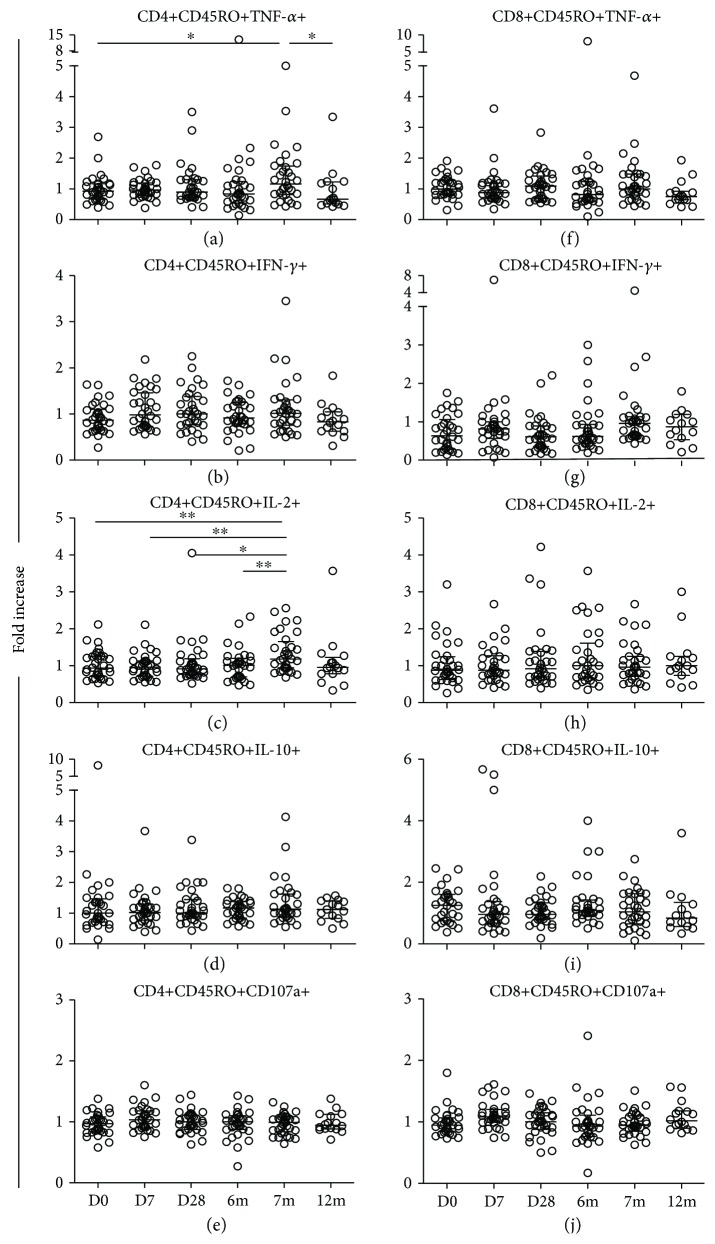
Fold increases in cytokine-producing and CD107a-expressing memory T cells in healthy control individuals. Fold increases in TNF-*α*, IFN-*γ*, IL-2, and IL-10 cytokine production, and CD107a expression, by memory CD4+ (a–e) and CD8+ (f–j) T cells in response to recombinant HBsAg by HIV− healthy individuals who were vaccinated with the standard dose regimen, compared before *in vivo* vaccination on day 0 (D0), and at day 7 (D7), day 28 (D28), month 6 (6m), month 7 (7m), and month 12 (12m) after vaccination. Medians are represented by thick, wide horizontal bars; 25–75% interquartile ranges are represented by thin, narrow bars. All *p* values < 0.05 were considered statistically significant. “∗” and “∗∗” stand for *p* < 0.05 and *p* < 0.01, respectively.

To evaluate cytokine-producing and CD107a-expressing memory T cell populations, CD45RO marker was used in the gating strategy, as described in Materials and Methods (Figures 2(a)–2(d)). The percentages of memory CD4+ or CD8+ T cells that produced TNF-*α*, IFN-*γ*, IL-2, and IL-10, or expressed CD107a in response to *in vitro* stimulation with HBsAg or medium alone, are presented in Supplementary Table (available here). The median fold increase of the percentages of cytokine-producing and CD107a-expressing memory CD4+ and CD8+ T cells between the study groups was compared between D0 as the baseline, D7, 1m, 2m (only for the four doses and four double doses groups), 6m, 7m, and 12m after vaccination. In the healthy control group (Figure 3), there were no statistical differences in the IFN-*γ*- (Figure 3(b)) and IL-10-producing (Figure 3(d)) or the CD107a-expressing (Figure 3(e)) memory CD4+ T cells between all the time points of the study. However, the fold increases of the TNF-*α*-producing memory CD4+ T cells (Figure 3(a)) at 7m after vaccination were significantly higher than those at prevaccination (D0, *p* = 0.042) and 12m (*p* = 0.02). The fold increases of the IL-2-producing memory CD4+ T cells (Figure 3(c)) at 7m after vaccination were statistically significantly higher than those at D0 (*p* = 0.008), D7 (*p* = 0.007), 1m (*p* = 0.027), and 6m (*p* = 0.009). In contrast to memory CD4+ T cells, no differences in the fold increase of cytokine production and expression of CD107a of memory CD8+ T cells (Figures 3(f)–3(j)) were observed at all the time points of the study. In all the study groups of the HIV+ individuals, there were no statistical differences in the median fold increases of cytokine production and expression of CD107a of the memory CD4+ and CD8+ T cells at any time point of the study (data not shown).

**Figure 4:**
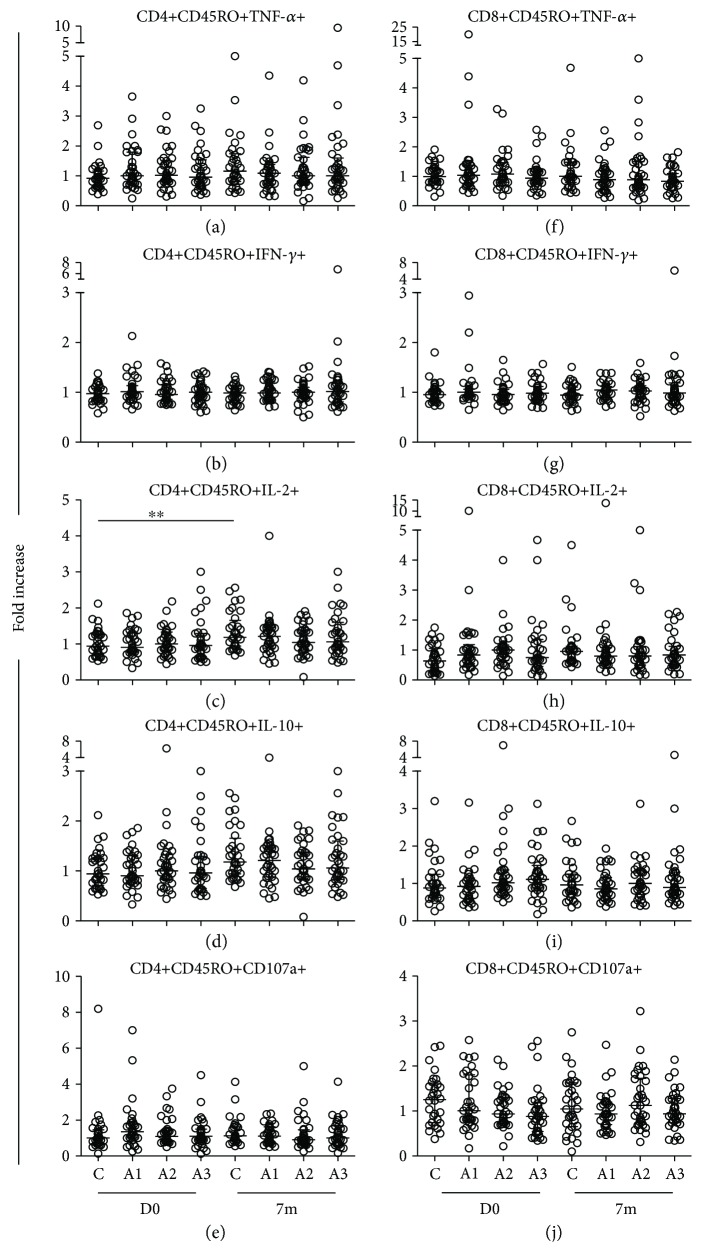
Comparison of fold increases in cytokine-producing and CD107a-expressing memory T cells between study groups. Fold increases in TNF-*α*, IFN-*γ*, IL-2, and IL-10 cytokine production, and CD107a expression, by memory CD4+ (a–e) and CD8+ (f–j) T cells in response to recombinant HBsAg between healthy controls (c), the standard dose group (A1), the four doses group (A2), and the four double doses group (A3) were compared before *in vivo* vaccination on day 0 (D0) and month 7 (7m) after vaccination. Medians are represented by thick, wide horizontal bars; 25–75% interquartile ranges are represented by thin, narrow bars. All *p* values < 0.05 were considered statistically significant. “∗∗” stands for *p* < 0.01.

**Figure 5:**
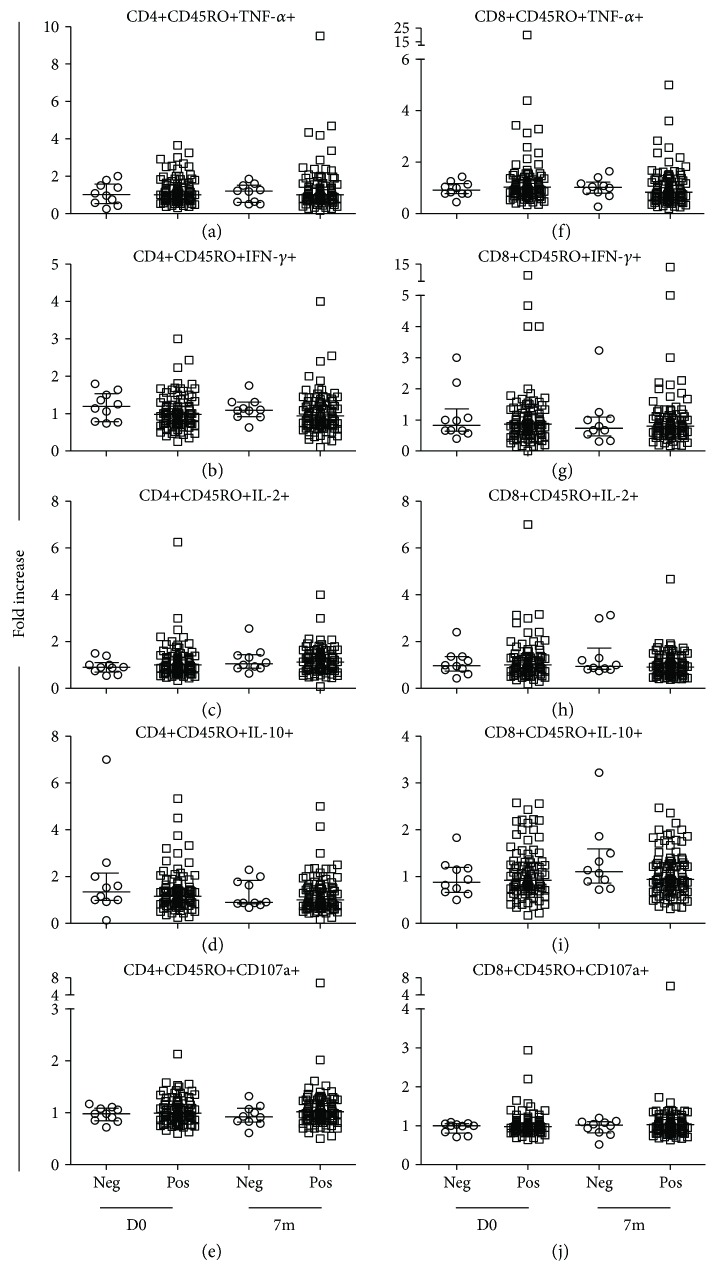
Fold increases in cytokine-producing and CD107a-expressing memory T cells between groups with regard to serological responsiveness to the vaccine. Fold increases in TNF-*α*, IFN-*γ*, IL-2, and IL-10 production, and CD107a expression, by memory CD4+ (a) and CD8+ (b) T cells in response to recombinant HBsAg between seronegative (Neg) and seropositive (Pos) individuals among all the HIV+ participants were compared before *in vivo* vaccination on day 0 (D0) and month 7 (7m) after vaccination. Medians are represented by thick, wide horizontal bars; 25–75% interquartile ranges are represented by thin, narrow bars.

**Figure 6:**
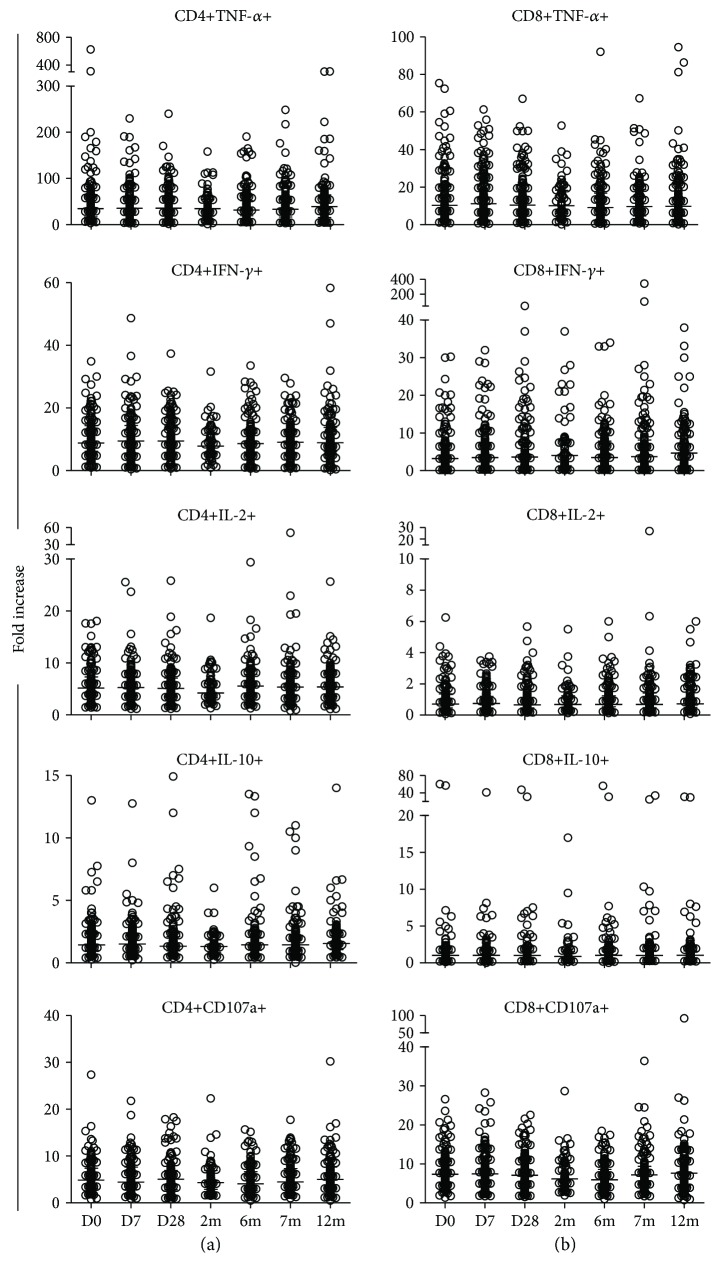
Fold increases in cytokine-producing and CD107a-expressing T cells in response to PHA stimulation. Fold increases in TNF-*α*, IFN-*γ*, IL-2, and IL-10 cytokine production, and CD107a expression, by CD4+ (a) and CD8+ (b) T cells in response to PHA stimulation by all participants, compared with regard to before *in vivo* vaccination on day 0 (D0) and at day 7 (D7), day 28 (D28), month 6 (6m), month 7 (7m), and month 12 (12m) after vaccination. There were no statistical differences in the responses between visits. Medians are represented by thick, wide horizontal bars; 25–75% interquartile ranges are represented by thin, narrow bars.

When compared between four groups at D0 and 7m, statistical analysis of the fold increases of cytokine-producing and CD107a-expressing memory CD4+ (Figures 4(a)–4(e)) and CD8+ (Figures 4(f)–4(j)) T cells did not demonstrate statistically significant differences between the groups. The data were further analyzed by comparing the median fold increases between the study groups. Based on the definition of responsiveness to HBV vaccination, which is generation of anti-HBsAg antibodies ≥ 10 IU/mL (levels presumptive for seroprotection) at 1 month after three doses of vaccination [35], and since we observed an increase in the cytokine-producing T cells at 7m after initial vaccination, as described above, the comparisons of the cytokine-producing and CD107a-expressing T cells between the study groups at the baseline and at 7m after first vaccination were selected. There were no statistical differences in the median fold increases of all the cytokine-producing and CD107a-expressing memory CD4+ (Figure 5(a)) and CD8+ (Figure 5(b)) T cells between the HBV vaccine serological nonresponders and the responders compared at D0 and 7m after vaccination. In response to positive control phytohaemagglutinin (PHA) stimulation, strong cytokine production and CD107a expression were observed in all the participants regardless of their HIV status. There were no differences in these responses between the groups at all the time points of the study (Figure 6).

## 4. Discussion

The effectiveness of HBV vaccination in HIV+ individuals has been broadly investigated in relation to humoral immune response. In contrast, the knowledge of cellular immune responses is much less well understood. This study focused on the cytokine production and expression of the degranulation marker of CD4+ and CD8+ T cells in response to *in vitro* recombination HBsAg stimulation in HIV+ individuals following different vaccination regimes as compared to those in healthy donors.

We found no statistical differences in the frequencies of cytokine production and CD107a expression of total or memory CD4+ or CD8+ T cells during the 12 months of study in all the three HIV+ groups. This was not because of T cell defects or technical problem during the assays since T cells from all the participants produced cytokines in response to PHA stimulation, demonstrating the functional activity of the cryopreserved PBMCs. However, the results showed an increase in the TNF-*α*-producing total and memory CD4+ T cells at 7m after vaccination in healthy controls compared to the HIV+ group, which received the same vaccination regimen (standard dose group). TNF-*α* has a direct effect on the proliferation of HBV-specific CTLs [36]. Upregulation of TNF-*α* of HBV-specific CTL abrogates gene expression and replication of HBV without killing infected hepatocytes [29, 37]. TNF-*α* also plays an important role in the inhibition of HBV-specific regulatory T cell-suppressive function [38]. Higher production of TNF-*α* of HBV-specific CD4+ T cells after vaccination in healthy individuals suggests that at the standard vaccination regimen, they might achieve better control of HBV replication than do HIV+ individuals.

We also found an increase in HBsAg-specific IL-2-producing memory CD4+ T cells at 7m after vaccination in the healthy control group. IL-2 has an effect on many immune cell types, especially lymphocytes for their differentiation and proliferation [39, 40]. IL-2 secretion by memory CD4+ T cells is essential for B cell differentiation into IgG-producing plasma cells [41]. In general, vaccination with protein antigens in humans induces antigen-specific T cells to produce IL-2 more dominantly than do other cytokines [42, 43]. The increase in IL-2 production in the healthy control group would be beneficial to these individuals against HBV infection. However, comparison of the IL-2-producing memory CD4+ T cells between all four groups at 7m after vaccination, which was the peak of the response in the healthy controls, did not show any statistical difference. In addition, no significant increases in the cytokine-producing T cells or memory T cells were observed at all the time points during the investigation in the HIV+ groups. This might be the result of little increases in these HBV-specific cells. Whether using HBsAg-derived peptide pools may improve the sensitivity of the assay will be of great interest. No statistical differences in the frequencies of polyfunctional T cells in response to recombinant HBsAg between healthy controls and HIV+ groups were found (data not shown). This might be due to invigorated responses observed in our study. It should be noted that the absolute CD4 T cell counts of the HIV+ groups in our study and their viral loads were in good levels. All of them were also receiving ART. This could be a reason that no profound differences were observed in this study. Furthermore, the HBV vaccine used in this study was highly immunogenic in our populations as reflected by the high seroconversion rate in all the three HIV+ study groups [18]. The percentage of serological responders to HBV vaccination in HIV+ adults receiving the standard vaccination regimen was almost as high as that achieved in non-HIV healthy adults, in addition to much higher seroconversion rate than that reported by other studies [13, 19, 44, 45]. Such effective vaccine might induce CMI responses to a nearly comparable level in all groups.

The correlation between cellular and humoral immunity in response to HBV vaccination is still controversial. We did not find any differences in cytokine production between serological responders and nonresponders among HIV+ individuals. Our results were similar to those reported in healthy individuals [46]. Nevertheless, the production of cytokines observed in serological nonresponders in our and other studies [46] may shed light on the induction of specific cellular immunity to control HBV infection in this population. This issue warrants further investigation.

## 5. Conclusion

This study suggests that the standard HBV vaccination schedule induces the TNF-*α*- and IL-2-producing CD4+ T cells at 7m after vaccination in healthy individuals, but that only the TNF-*α*-producing CD4+ T cells were better than those of the HIV+ individuals who received the same vaccination regimen. Therefore, increased doses and/or frequencies may be beneficial for the HIV+ populations. Although further investigations are needed in order to explore the mechanisms that may contribute to protective CMI, the information from this study may provide valuable perception for future vaccine design to improve cellular immune responses to HBV vaccination for HIV+ populations.

## Supplementary Materials

Supplementary Materials Supplementary Table: the median and interquartile range of the percentages of cytokine-producing and CD107a-expressing memory CD4+ and CD8+ T cells in response to *in vitro* stimulation with HBsAg or medium alone (unstimulated).
