# Supplementary material for: Hepatitis B Vaccination Induced TNF-α- and IL-2-Producing T Cell Responses in HIV− Healthy Individuals Higher than in HIV+ Individuals Who Received the Same Vaccination Regimen
Source: J Immunol Res. 2018 Feb 27;2018:8350862. doi: 10.1155/2018/8350862 (PMC5848135; doi:10.1155/2018/8350862)
Supplement: Supplementary Materials — Supplementary Table: the median and interquartile range of the percentages of cytokine-producing and CD107a-expressing memory CD4+ and CD8+ T cells in response to in vitro stimulation with HBsAg or medium alone (unstimulated). [file 8350862.f1.doc]

**Supplementary Table. The median and interquartile range of the percentages of cytokine-producing and CD107a-expressing memory CD4+ and CD8+ T cells in response to *in vitro* stimulation with HBsAg or medium alone (unstimulated).**

| Group | T cell population | Antigen Stimulation | Cytokines | Percentag of cytokine-producing memory T cells | | | | | | |
| --- | --- | --- | --- | --- | --- | --- | --- | --- | --- | --- |
| Day 0 | Day 7 | Day 28 | 2 months | 6 months | 7 months | 12 months |
| Healthy control | Memory CD4+ | Unstimulated | TNFα | 0.26 (0.20-0.41) | 0.26 (0.26-0.46) | 0.28 (0.21-0.46) |  | 0.31 (0.15-0.52) | 0.27 (0.14-0.51) | 0.24 (0.17-0.55) |
|  |  | HBs Ag |  | 0.27 (0.16-0.35) | 0.27 (0.16-0.41) | 0.30 (0.17-0.51) |  | 0.28 (0.16-0.47) | 0.31 (0.16-0.48) | 0.22 (0.11-0.35) |
|  |  | Unstimulated | IFNγ | 0.16 (0.12-0.24) | 0.16 (0.12-0.25) | 0.20 (0.14-0.25) |  | 0.17 (0.12-0.23) | 0.13 (0.10-0.23) | 0.20 (0.15-0.29) |
|  |  | HBs Ag |  | 0.15 (0.10-0.22) | 0.16 (0.11-0.26) | 0.19 (0.14-0.26) |  | 0.15 (0.10-0.25) | 0.15 (0.11-0.22) | 0.17 (0.11-0.28) |
|  |  | Unstimulated | IL2 | 0.17 (0.14-0.25) | 0.19 (0.13-0.26) | 0.19 (0.15-0.23) |  | 0.20 (0.14-0.25) | 0.17 (0.12-0.25) | 0.18 (0.12-0.26) |
|  |  | HBs Ag |  | 0.19 (0.14-0.21) | 0.19 (0.14-0.23) | 0.19 (0.14-0.26) |  | 0.18 (0.14-0.22) | 0.22 (0.16-0.29) | 0.16 (0.13-0.22) |
|  |  | Unstimulated | IL10 | 0.14 (0.09-0.20) | 0.16 (0.10-0.21) | 0.16 (0.09-0.22) |  | 0.17 (0.08-0.24) | 0.15 (0.09-0.22) | 0.16 (0.07-0.24) |
|  |  | HBs Ag |  | 0.18 (0.07-0.23) | 0.15 (0.08-0.23) | 0.17 (0.10-0.23) |  | 0.17 (0.09-0.24) | 0.16 (0.11-0.26) | 0.19 (0.10-0.28) |
|  |  | Unstimulated | CD107a | 1.71 (1.49-2.35) | 2.31 (1.70-2.71) | 2.21 (1.85-2.73) |  | 1.90 (1.40-2.37) | 1.80 (1.36-2.27) | 2.58 (2.04-4.04) |
|  |  | HBs Ag |  | 1.87 (1.41-2.29) | 2.24 (1.71-2.76) | 2.23 (1.60-2.84) |  | 1.86 (1.43-2.21) | 1.70 (1.05-2.19) | 2.38 (1.95-4.10) |
|  | Memory CD8+ | Unstimulated | TNFα | 0.83 (0.55-1.50) | 0.98 (0.45-1.39) | 0.80 (0.44-1.27) |  | 0.83 (0.47-1.55) | 0.69 (0.40-1.26) | 0.94 (0.72-1.31) |
|  |  | HBs Ag |  | 0.90 (0.67-1.31) | 0.81 (0.47 -1.20) | 0.83 (0.47-1.04) |  | 0.74 (0.37-1.48) | 0.72 (0.43-1.31) | 0.68 (0.39-1.08) |
|  |  | Unstimulated | IFNγ | 0.18 (0.13-0.25) | 0.20 (0.12-0.33) | 0.23 (0.17-0.40) |  | 0.24 (0.14-0.34) | 0.19 (0.13-0.53) | 0.20 (0.10-0.30) |
|  |  | HBs Ag |  | 0.13 (0.04-0.27) | 0.13 (0.09-0.27) | 0.11 (0.07-0.28) |  | 0.13 (0.07-0.37) | 0.19 (0.08-0.35) | 0.11 (0.05-0.28) |
|  |  | Unstimulated | IL2 | 0.18 (0.11-0.23) | 0.15 (0.10-0.34) | 0.19 (0.11-0.29) |  | 0.19 (0.10-0.27) | 0.17 (0.10-0.29) | 0.16 (0.10-0.28) |
|  |  | HBs Ag |  | 0.16 (0.10-0.26) | 0.17 (0.10-0.29) | 0.17 (0.11-0.32) |  | 0.18 (0.09-0.30) | 0.17 (0.10-0.23) | 0.15 (0.12-0.23) |
|  |  | Unstimulated | IL10 | 0.18 (0.14-0.25) | 0.23 (0.14-0.38) | 0.26 (0.15-0.33) |  | 0.22 (0.13-0.34) | 0.24 (0.15-0.35) | 0.19 (0.11-0.25) |
|  |  | HBs Ag |  | 0.27 (0.17-0.33) | 0.22 (0.15-0.38) | 0.22 (0.14-0.36) |  | 0.24 (0.18-0.30) | 0.22 (0.13-0.37) | 0.17 (0.08-0.26) |
|  |  | Unstimulated | CD107a | 3.64 (2.71-4.46) | 3.89 (2.58-4.78) | 4.04 (2.99-4.87) |  | 3.51 (2.52-4.90) | 2.78 (2.35-4.28) | 5.47 (3.06-7.12) |
|  |  | HBs Ag |  | 3.25 (2.76-4.83) | 4.04 (3.03-5.61) | 3.73 (2.79-5.64) |  | 3.32 (2.67-3.90) | 2.83 (2.16-4.38) | 4.95 (3.93-7.64) |
| Standard dose | Memory CD4+ | Unstimulated | TNFα | 0.19 (0.10−0.39) | 0.20 (0.11−0.44) | 0.18 (0.13−0.30) |  | 0.23 (0.14−0.53) | 0.22 (0.11−0.46) | 0.24 (0.13−0.58) |
|  |  | HBs Ag |  | 0.19 (0.11−0.56) | 0.20 (0.08−0.40) | 0.19 (0.11−0.46) |  | 0.26 (0.10−0.47) | 0.22 (0.14−0.46) | 0.26 (0.14−0.47) |
|  |  | Unstimulated | IFNγ | 0.16 (0.13−0.25) | 0.18 (0.11−0.25) | 0.18 (0.11−0.25) |  | 0.17 (0.13−0.34) | 0.21 (0.12−0.33) | 0.17 (0.11−0.26) |
|  |  | HBs Ag |  | 0.16 (0.10−0.25) | 0.15 (0.11−0.25) | 0.17 (0.11−0.29) |  | 0.23 (0.11−0.30) | 0.16 (0.13−0.24) | 0.18 (0.13−0.27) |
|  |  | Unstimulated | IL2 | 0.18 (0.14−0.28) | 0.18 (0.13−0.24) | 0.19 (0.15−0.24) |  | 0.18 (0.13−0.25) | 0.17 (0.11−0.24) | 0.17 (0.14−0.26) |
|  |  | HBs Ag |  | 0.17 (0.12−0.26) | 0.18 (0.14−0.22) | 0.19 (0.15−0.25) |  | 0.18 (0.12−0.25) | 0.20 (0.14−0.25) | 0.19 (0.13−0.25) |
|  |  | Unstimulated | IL10 | 0.17 (0.10−0.22) | 0.16 (0.10−0.20) | 0.15 (0.12−0.22) |  | 0.14 (0.10−0.20) | 0.17 (0.11−0.24) | 0.16 (0.10−0.23) |
|  |  | HBs Ag |  | 0.21 (0.13−0.27) | 0.17 (0.12−0.28) | 0.20 (0.14−0.27) |  | 0.20 (0.12−0.26) | 0.18 (0.13−0.26) | 0.19 (0.13−0.31) |
|  |  | Unstimulated | CD107a | 1.58 (1.31−2.24) | 1.73 (1.39−2.19) | 1.86 (1.45−2.61) |  | 2.52 (1.88−3.33) | 1.82 (1.47−2.35) | 1.70 (1.37−2.26) |
|  |  | HBs Ag |  | 1.70 (1.24−2.40) | 1.80 (1.44−2.13) | 1.81 (1.55−2.30) |  | 2.55 (1.95−3.25) | 1.84 (1.42−2.67) | 1.68 (1.27−2.55) |
|  | Memory CD8+ | Unstimulated | TNFα | 0.46 (0.19−0.72) | 0.48 (0.18−0.87) | 0.42 (0.20−0.74) |  | 0.51 (0.23−0.87) | 0.54 (0.22−0.92) | 0.41 (0.17−0.85) |
|  |  | HBs Ag |  | 0.43 (0.19−0.82) | 0.38 (0.14−0.88) | 0.38 (0.22−0.68) |  | 0.45 (0.22−0.76) | 0.35 (0.23−0.78) | 0.44 (0.23−0.88) |
|  |  | Unstimulated | IFNγ | 0.21 (0.11−0.65) | 0.20 (0.09−0.64) | 0.20 (0.12−0.63) |  | 0.19 (0.09−0.47) | 0.25 (0.13−0.57) | 0.20 (0.08−0.67) |
|  |  | HBs Ag |  | 0.20 (0.08−0.50) | 0.12 (0.08−0.52) | 0.17 (0.10−0.74) |  | 0.18 (0.07−0.48) | 0.16 (0.07−0.43) | 0.16 (0.07−0.61) |
|  |  | Unstimulated | IL2 | 0.20 (0.13−0.32) | 0.18 (0.13−0.37) | 0.19 (0.12−0.33) |  | 0.20 (0.10−0.32) | 0.17 (0.13−0.35) | 0.19 (0.10−0.27) |
|  |  | HBs Ag |  | 0.16 (0.11−0.36) | 0.17 (0.13−0.31) | 0.19 (0.12−0.34) |  | 0.17 (0.10−0.31) | 0.18 (0.08−0.35) | 0.18 (0.09−0.32) |
|  |  | Unstimulated | IL10 | 0.12 (0.13−0.38) | 0.18 (0.14−0.34) | 0.24 (0.13−0.35) |  | 0.21 (0.10−0.33) | 0.21 (0.14−0.41) | 0.23 (0.13−0.41) |
|  |  | HBs Ag |  | 0.24 (0.12−0.43) | 0.19 (0.11−0.33) | 0.24 (0.12−0.42) |  | 0.19 (0.10−0.37) | 0.22 (0.13−0.35) | 0.21 (0.14−0.40) |
|  |  | Unstimulated | CD107a | 2.84 (2.04−4.07) | 3.19 (2.17−4.42) | 3.30 (2.55−4.67) |  | 3.56 (2.78−5.68) | 3.14 (2.51−4.33) | 2.95 (2.04−3.63) |
|  |  | HBs Ag |  | 2.90 (2.12−4.32) | 2.93 (2.31−4.27) | 3.34 (2.37−4.33) |  | 3.71 (2.77−5.80) | 3.22 (2.43−4.44) | 2.90 (1.83−4.28) |
| Four doses | Memory CD4+ | Unstimulated | TNFα | 0.26 (0.15−0.45) | 0.21 (0.15−0.40) | 0.28 (0.16−0.44) | 0.27 (0.17−0.41) | 0.30 (0.19−0.48) | 0.28 (0.18−0.52) | 0.22 (0.17−0.40) |
|  |  | HBs Ag |  | 0.25 (0.13−0.50) | 0.29 (0.15−0.45) | 0.28 (0.12−0.40) | 0.23 (0.17−0.39) | 0.30 (0.18−0.51) | 0.32 (0.18−0.49) | 0.27 (0.20−0.42) |
|  |  | Unstimulated | IFNγ | 0.18 (0.15−0.21) | 0.19 (0.11−0.30) | 0.18 (0.14−0.25) | 0.18 (0.13−0.28) | 0.20 (0.13−0.27) | 0.19 (0.14−0.26) | 0.17 (0.13−0.25) |
|  |  | HBs Ag |  | 0.17 (0.12−0.25) | 0.12 (0.13−0.25) | 0.17 (0.12−0.26) | 0.16 (0.13−0.23) | 0.17 (0.14−0.23) | 0.17 (0.13−0.25) | 0.16 (0.13−0.23) |
|  |  | Unstimulated | IL2 | 0.18 (0.12−0.21) | 0.18 (0.13−0.26) | 0.18 (0.14−0.25) | 0.20 (0.15−0.26) | 0.16 (0.13−0.23) | 0.17 (0.13−0.26) | 0.19 (0.13−0.22) |
|  |  | HBs Ag |  | 0.18 (0.12−0.23) | 0.19 (0.12−0.29) | 0.16 (0.14−0.24) | 0.16 (0.13−0.24) | 0.18 (0.13−0.24) | 0.20 (0.13−0.27) | 0.20 (0.15−0.25) |
|  |  | Unstimulated | IL10 | 0.15 (0.08−0.19) | 0.14 (0.08−0.19) | 0.14 (0.09−0.21) | 0.13 (0.10−0.19) | 0.16 (0.08−0.19) | 0.13 (0.07−0.22) | 0.14 (0.08−0.20) |
|  |  | HBs Ag |  | 0.15 (0.11−0.22) | 0.17 (0.09−0.26) | 0.18 (0.11−0.26) | 0.15 (0.09−0.24) | 0.16 (0.11−0.22) | 0.13 (0.07−0.19) | 0.15 (0.09−0.22) |
|  |  | Unstimulated | CD107a | 1.90 (1.38−3.06) | 1.97 (1.51−2.69) | 2.02 (1.48−2.88) | 2.33 (1.76−2.86) | 2.62 (1.85−3.42) | 2.29 (1.66−2.92) | 2.33 (1.59−2.58) |
|  |  | HBs Ag |  | 1.85 (1.40−2.51) | 2.08 (1.39−2.46) | 1.98 (1.45−2.79) | 2.20 (1.55−2.69) | 2.48 (1.90−3.31) | 2.27 (1.78−2.84) | 2.11 (1.61−2.79) |
|  | Memory CD8+ | Unstimulated | TNFα | 0.29 (0.20−0.74) | 0.35 (0.21−0.80) | 0.36 (0.22−0.80) | 0.38 (0.28−0.90) | 0.52 (0.26−0.86) | 0.45 (0.24−1.02) | 0.51 (0.20−0.79) |
|  |  | HBs Ag |  | 0.38 (0.25−0.79) | 0.39 (0.27−0.80) | 0.39 (0.20−0.75) | 0.44 (0.21−0.86) | 0.48 (0.27−1.09) | 0.43 (0.24−0.85) | 0.41 (0.21−0.76) |
|  |  | Unstimulated | IFNγ | 0.18 (0.07−0.38) | 0.14 (0.08−0.32) | 0.20 (0.05−0.49) | 0.16 (0.09−0.31) | 0.11 (0.06−0.28) | 0.12 (0.05−0.30) | 0.12 (0.04−0.24) |
|  |  | HBs Ag |  | 0.12 (0.05−0.36) | 0.13 (0.06−0.30) | 0.14 (0.06−0.30) | 0.14 (0.06−0.24) | 0.11 (0.04−0.26) | 0.09 (0.04−0.22) | 0.07 (0.04−0.23) |
|  |  | Unstimulated | IL2 | 0.14 (0.10−0.24) | 0.18 (0.11−0.25) | 0.13 (0.09−0.27) | 0.19 (0.12−0.25) | 0.16 (0.11−0.26) | 0.15 (0.09−0.22) | 0.13 (0.10−0.18) |
|  |  | HBs Ag |  | 0.16 (0.11−0.28) | 0.15 (0.11−0.24) | 0.15 (0.10−0.23) | 0.18 (0.13−0.27) | 0.16 (0.10−0.22) | 0.15 (0.08−0.25) | 0.14 (0.08−0.24) |
|  |  | Unstimulated | IL10 | 0.25 (0.14−0.36) | 0.22 (0.12−0.30) | 0.22 (0.14−0.33) | 0.18 (0.14−0.32) | 0.18 (0.12−0.28) | 0.17 (0.09−0.26) | 0.14 (0.10−0.25) |
|  |  | HBs Ag |  | 0.23 (0.12−0.40) | 0.22 (0.14−0.43) | 0.23 (0.14−0.35) | 0.20 (0.15−0.29) | 0.21 (0.12−0.33) | 0.17 (0.12−0.28) | 0.18 (0.12−0.29) |
|  |  | Unstimulated | CD107a | 3.16 (1.97−4.80) | 3.18 (1.87−4.88) | 3.16 (2.14−4.64) | 3.50 (2.45−5.74) | 4.13 (3.01−5.51) | 3.34 (2.77−4.91) | 3.45 (2.16−4.49) |
|  |  | HBs Ag |  | 2.86 (2.03−4.75) | 3.00 (2.01−4.45) | 3.13 (2.53−4.98) | 3.69 (2.54−5.25) | 4.02 (2.99−5.98) | 3.38 (2.50−5.13) | 3.51 (2.58−4.49) |
| Four double doses | Memory CD4+ | Unstimulated | TNFα | 0.28 (0.18−0.50) | 0.28 (0.20−0.54) | 0.27 (0.16−0.55) | 0.32 (0.18−0.43) | 0.27 (0.18−0.44) | 0.26 (0.18−0.57) | 0.24 (0.17−0.48) |
|  | HBs Ag |  | 0.30 (0.19−0.44) | 0.24 (0.19−0.46) | 0.25 (0.14−0.44) | 0.30 (0.19−0.53) | 0.29 (0.18−0.48) | 0.29 (0.20−0.52) | 0.29 (0.20−0.45) |
|  |  | Unstimulated | IFNγ | 0.18 (0.12−0.25) | 0.20 (0.14−0.29) | 0.14 (0.11−0.26) | 0.18 (0.15−0.25) | 0.20 (0.15−0.25) | 0.18 (0.12−0.25) | 0.17 (0.11−0.25) |
|  |  | HBs Ag |  | 0.18 (0.12−0.24) | 0.18 (0.11−0.26) | 0.15 (0.10−0.25) | 0.17 (0.12−0.25) | 0.19 (0.11−0.27) | 0.17 (0.12−0.22) | 0.16 (0.10−0.24) |
|  |  | Unstimulated | IL2 | 0.16 (0.13−0.23) | 0.19 (0.13−0.27) | 0.16 (0.10−0.22) | 0.17 (0.12−0.23) | 0.17 (0.13−0.23) | 0.16 (0.13−0.23) | 0.18 (0.14−0.25) |
|  |  | HBs Ag |  | 0.17 (0.13−0.23) | 0.19 (0.14−0.27) | 0.16 (0.11−0.21) | 0.17 (0.13−0.26) | 0.18 (0.14−0.21) | 0.19 (0.14−0.29) | 0.18 (0.13−0.29) |
|  |  | Unstimulated | IL10 | 0.15 (0.09−0.19) | 0.08 (0.13−0.21) | 0.14 (0.08−0.22) | 0.16 (0.09−0.22) | 0.15 (0.08−0.18) | 0.15 (0.07−0.20) | 0.14 (0.08−0.18) |
|  |  | HBs Ag |  | 0.17 (0.09−0.21) | 0.14 (0.08−0.21) | 0.15 (0.10−0.23) | 0.15 (0.10−0.19) | 0.16 (0.12−0.20) | 0.15 (0.07−0.21) | 0.14 (0.10−0.21) |
|  |  | Unstimulated | CD107a | 1.82 (1.63−2.61) | 2.35 (1.55−2.96) | 2.24 (1.50−3.23) | 2.36 (1.71−3.36) | 2.43 (1.97−3.71) | 2.34 (1.72−3.32) | 2.20 (1.56−2.90) |
|  |  | HBs Ag |  | 1.91 (1.55−2.53) | 2.23 (1.69−2.73) | 1.93 (1.53−2.54) | 2.17 (1.76−2.95) | 2.30 (1.94−3.14) | 2.50 (1.94−3.16) | 2.08 (1.46−2.79) |
|  | Memory CD8+ | Unstimulated | TNFα | 0.62 (0.23−0.91) | 0.54 (0.34−0.90) | 0.40 (0.27−0.91) | 0.57 (0.33−0.98) | 0.53 (0.31−1.00) | 0.60 (0.36−1.02) | 0.49 (0.23−0.90) |
|  |  | HBs Ag |  | 0.43 (0.28−0.74) | 0.45 (0.23−0.67) | 0.35 (0.25−0.82) | 0.55 (0.38−0.85) | 0.52 (0.29−0.88) | 0.50 (0.26−0.77) | 0.44 (0.24−0.93) |
|  |  | Unstimulated | IFNγ | 0.13 (0.06−0.41) | 0.13 (0.07−0.33) | 0.17 (0.07−0.42) | 0.13 (0.07−0.40) | 0.16 (0.07−0.35) | 0.15 (0.07−0.30) | 0.13 (0.15−0.28) |
|  |  | HBs Ag |  | 0.12 (0.04−0.37) | 0.11 (0.03−0.25) | 0.14 (0.04−0.35) | 0.15 (0.04−0.31) | 0.13 (0.04−0.24) | 0.14 (0.03−0.21) | 0.10 (0.03−0.25) |
|  |  | Unstimulated | IL2 | 0.17 (0.10−0.22) | 0.16 (0.12−0.20) | 0.14 (0.11−0.25) | 0.16 (0.10−0.22) | 0.18 (0.12−0.21) | 0.14 (0.09−0.20) | 0.16 (0.11−0.20) |
|  |  | HBs Ag |  | 0.18 (0.10−0.26) | 0.14 (0.09−0.19) | 0.16 (0.11−0.25) | 0.17 (0.11−0.23) | 0.14 (0.10−0.21) | 0.13 (0.09−0.21) | 0.13 (0.09−0.19) |
|  |  | Unstimulated | IL10 | 0.20 (0.12−0.38) | 0.17 (0.14−0.29) | 0.19 (0.11−0.27) | 0.20 (0.13−0.27) | 0.18 (0.10−0.27) | 0.19 (0.11−0.28) | 0.18 (0.09−0.27) |
|  |  | HBs Ag |  | 0.20 (0.10−0.28) | 0.13 (0.08−0.22) | 0.17 (0.09−0.25) | 0.18 (0.11−0.33) | 0.15 (0.09−0.28) | 0.16 (0.09−0.25) | 0.17 (0.10−0.24) |
|  |  | Unstimulated | CD107a | 3.12 (2.39−4.67) | 3.68 (2.57−5.28) | 3.20 (2.45−5.91) | 4.15 (3.14−5.29) | 4.24 (2.85−5.87) | 3.86 (2.66−5.30) | 3.55 (2.39−4.74) |
|  |  | HBs Ag |  | 3.34 (2.22−4.25) | 3.47 (2.67−5.18) | 3.26 (2.30−5.52) | 4.16 (3.07−6.00) | 4.20 (2.76−5.22) | 4.06 (2.91−5.89) | 3.62 (2.62−4.87) |
